# Free Radical Scavenging Activity of *Calotropis gigantea* on Streptozotocin-Induced Diabetic Rats

**DOI:** 10.4103/0250-474X.59542

**Published:** 2009

**Authors:** N. R. Rathod, I. Raghuveer, H. R. Chitme, R. Chandra

**Affiliations:** Department of Pharmacology, H. S. K. College of Pharmacy, Bagalkot-587101, India; 1 Institute of Pharmacy, Bundelkhand University, Jhansi-284128, India; 2 Oman Medical College Azaiba, Muscat, Sultanate of Oman, India; 3 DR. B. R. Ambedkar Centre for Biomedical Research, New Delhi-110007, India

**Keywords:** *Calotropis gigantea*, diabetes, oxidative stress, *swarnabhasma*

## Abstract

*Swarnabhasma*, an Ayurvedic preparation containing *Calotropis gigantea* R. Br. (Asclepiadaceae) is extensively used by Ayurvedic physicians for treatment of diabetes mellitus, bronchial asthma, rheumatoid arthritis and nervous disorders. In the present study, we report the effect of chloroform extracts of *Calotropis gigantea* leaf and flower on free radical scavenging activity, and lipid profile in streptozotozin-induced diabetic rats. The lipid peroxidation, superoxide dismutase, and catalase were measured in liver homogenate and serum glutamic pyruvic transaminase, serum glutamic oxaloacetic transaminase, alkaline phosphatase, lipid profile were measured in blood serum. Administration of single dose of streptozotozin (55 mg/kg, i.p.) caused significant increases in lipid peroxidation, serum glutamic pyruvic transaminase, serum glutamic oxaloacetic transaminase, alkaline phosphatase, cholesterol and triglyceride levels, while superoxide dismutase and catalase levels were significantly decreased. Further, administration of chloroform extracts of *Calotropis gigantea* leaf and flower to streptozotocin-induced diabetes rats at a dose of 10, 20 and 50 mg/kg orally for 27 d lead to a significant decrease in lipid peroxidation, serum glutamic pyruvic transaminase, serum glutamic oxaloacetic transaminase, alkaline phosphatase, cholesterol and triglyceride levels. Consequently, superoxide dismutase and catalase levels were significantly increased. Glibenclamide was used as a positive control (10 mg/kg). It was observed that the effect of chloroform extracts of *Calotropis gigantea* on alkaline phosphatase, cholesterol, superoxide dismutase, serum glutamic pyruvic transaminase, serum glutamic oxaloacetic transaminase, levels are comparable to that of those produced by the positive control.

Type 2 diabetes is associated with increased oxidative stress. Free radicals are continuously produced in the body as the result of normal metabolic processes and interaction with environmental stimuli. Under physiological conditions, a wide range of antioxidant defenses protect against adverse effects of free radical production *in vivo*[1]. Oxidative stress results from an imbalance between radical production and reduced activity of antioxidant defenses or both these phenomena. Hyperglycemia causes release of tissue damaging reactive oxygen species (ROS) that disturbs balance between radical production and protective antioxidant defense[2]. It has been proposed that streptozotocin (STZ) acts as a diabetogenic agent owing to its ability to destroy pancreatic β-cells, possibly by a free radical mechanism[3,4]. The level of lipid peroxidation in cell is controlled by various cellular defense mechanisms consisting of enzymatic and nonenzymatic scavenging systems which are altered in diabetes[5]. Moreover, disturbances of antioxidant defense systems in diabetes showed alteration in antioxidant enzyme levels, such as superoxide dismutase (SOD), catalase (CAT), glutathione peroxidase (GPx) and glutathione reductase (GR) along with impaired glutathione (GSH) metabolism[6]. Chemical with antioxidant properties and free radical scavengers may help in the regeneration of β-cell and protect pancreatic islets against cytotoxic effect of STZ[7]. Antioxidants provide protection in living organisms from damage caused by uncontrolled production of ROS concomitant lipid peroxidation, protein damage and DNA strand breaking. Ethno medical literature contains a large number of plants including,*Calotropis gigantea* R. Br (Asclepiadaceae) that can be used against diseases, like diabetes, atherosclerosis, ischeamic heart disease, disorders induced by free radicals and other reactive oxygen species[8]. Hence, the present study was under taken to explore *Calotropis gigantea* R. Br. (Asclepiadaceae) free radical scavenging activity in STZ-induced diabetic rats. In recent years, considerable focus has been given to an intensive search for novel type of antioxidants from numerous plant materials[9]. The management of diabetes without any side effects is still a challenge to the medical system. There is an increasing demand by patients for natural antidiabetic products, because insulin and oral hypoglycemic drugs possess undesirable side effects[10]. Plants endowed of antidiabetic properties provide useful sources for the development of drugs in the treatment of diabetes mellitus from ancient times; *Swarnabhasma*, an Ayurvedic preparation containing *Calotropis gigantea* R. Br (Asclepiadaceae) is extensively used by Ayurvedic physicians for treatment of disorders such as diabetes mellitus, bronchial asthma, rheumatoid arthritis, and nervous disorders[8]. Similar studies on another species *Calotropis procera* reported antioxidant activity against alloxan-induced diabetes in rats[11].

The roots of *Calotropis gigantea* have been used in leprosy, eczema, syphilis, elephantiasis, ulceration, antidiarrhoeal, and cough in the Indian system of traditional medicine. The following activities have been reported for *Calotropis gigantea;* prevention of insulin resistance[12], hepatoprotective[13], antidiarrhoeal[14], antipyretic and analgesic[15,16], antiinflammatory[17], analgesic activity in Eddy's hot plate, and acetic acid-induced writhings[18] and wound healing activity[19]. The milky juice of *Calotropis gigantea* has been reported as a violent purgative and gastrointestinal irritant and used for inducing abortion[20]. The alcohol extract of the flower of *Calotropis gigantea* reported analgesic activity in chemical and thermal models in mice[21]. The crude latex extract exhibited strong proteolytic activity, hydrolyses casein, human fibrinogen and crude fibrin clot in a dose-dependent manner[22].

Previously isolated classes of constituents include glycosides, proteases, 3'-methylbutanoates of amyrin, and taraxasterol from *Calotropis gigantea*[23], 19-nor- and 18,20-epoxy cardenolides from the leaves of *Calotropis gigantea*[24], stigmasterol and ß-sitosterol from methanol extract of root bark of *Calotropis gigantea*[25] and a new flavonol trisaccharide[26]. Autodigestion of two cystein proteinases, calotropins DI and DII isolated from the latex of *Calotropis gigantea* was studied at 37° (pH 7.5) in the presence of an activated agent; calotropin DI is more susceptible to autodigestion than calotropin DII[27]. The three-dimensional structure of sulfhydryl protease calotropin DI from *Calotropis gigantea* closely resembled those found in the sulfhydryl proteases papain and actinidin[28].

## MATERIALS AND METHODS

Streptozotocin was purchased from Sigma Chemical Co St Louis, USA. Petroleum ether (40-60°), chloroform, ethyl acetate, methanol purchased from Nice Pvt Ltd, India, and other chemical are purchased from Sigma and HiMedia Laboratories Pvt Ltd, Mumbai, India.

### Preparation of plant:

*Calotropis gigantea* leaf and flower were collected from widely growing plants from the region of North Karnataka in the months of Sept-Oct 2005. Plant was authenticated at Department of Botany, B. V. V. Sangh's Science College, Bagalkot. An voucher specimen (BSC/BOT/04/09) was deposited in the same Institute.

The plant material was shade-dried and uniformly powdered by passing through the sieve #44 subjected to hot continuous solvent extraction with petroleum ether (40-60°) to defat the preparation, followed by chloroform, ethyl acetate and methanol for 24 h cycle. The percentage of extract yield leaf and flower 1.32, 1.75, 0.87, 4.45 and 3.45, 2.15, 1.23, 2.10 was calculated in terms of dried weight. The solvent was distilled off, and excess solvent was completely removed by using rotary flash evaporator to get semisolid mass, and dried in lyophilizer (Mini Lyotrap, Serial No. J8199/5, LET Scientific LTD, UK). These extracts were formulated as suspension in distilled water using 5% Tween-80 as a suspending agent[29]. When different extracts were tested for hypoglycemic activity on normal rats, the chloroform extract was found to be active, the lowest and safe dose was selected based on the previous studies[12,14,18,20], whereas other extracts were found to be inactive. Therefore, the chloroform extract of *Calotropis gigantea* leaf and flower were selected for the present study. Henceforth, chloroform extracts of *Calotropis gigantea* leaf and flower refers to *Calotropis gigantea* extracts.

Wistar rats (150-200 g) were used for this study. They were kept in the animal house for 1 week for proper acclimatization before starting the experiment under controlled condition of illumination (12 h light/dark), and temperature 20-25°. They were housed under ideal laboratory conditions, maintained on standard pellet diet (Lipton rat feed Ltd, Pune), and water *ad libitum* throughout the experimental period. The experimental study was approved by the Institutional Animal's Ethics Committee (HSKCP/IAEC/Clear/2004-05).

### Induction of diabetes in rats:

The STZ was dissolved in citrate buffer (pH 4.5), and rats were made diabetic by injection of a single dose of STZ (55 mg/kg, i.p). They were given 5% of glucose in drinking water for the first 24 h to encounter any initial hypoglycemia. On the 3rd d animals were checked for serum blood glucose levels and those with more than 300 mg/dl were used in the study[30,31]. In our study, a total 54 rats (48 diabetic surviving rats, 6 normal rats) were used. These rats were randomly divided into 9 groups of six rats, after induction of STZ diabetes. Group No.1 (diabetic control) received distilled water in 5% Tween-80. Group No. 2 received glibenclamide (positive control) at an oral dose (10 mg/kg). Group No. 3 (normal) received distilled water in 5% Tween-80. Group No. 4, 5 and 6 received chloroform extract of *Calotropis gigantea* leaf (10, 20 and 50 mg/kg). Group No. 7, 8 and 9 received chloroform extract of *Calotropis gigantea* flower oral dose (10, 20 and 50 mg/kg) for 27 d, respectively.

### Preparation of liver homogenate:

The liver was weighed and 10% liver homogenate was prepared with 0.1 M phosphate buffer (pH 7.0) after centrifugation at 1000 rpm for 15 min. The supernatant was used to measure protein, lipid peroxidation (LPO), SOD, and CAT.

### Biochemical evaluations:

The blood serum, SGPT, SGOT, alkaline phosphatase (Alk Phos), cholesterol, and triglyceride were estimated by using commercial diagnostic kit, (Tecodiagnostics, USA) on star-21plus semi-autoanalyser.

### Protein content:

The protein content was estimated using a previously reported method[32]. Liver homogenate was mixed (1.0 ml) with 2.25 ml of 90% alcohol, and centrifuged at 3000 rpm for 10 min. The supernatant was discarded and the precipitate was dissolved in 1 ml of 0.1N NaoH, to which 1 ml of alkaline mixture was added, set aside for 10 min, 0.5 ml of folin reagent was added and again set aside for 10 min for complete color development. The absorbance was measured at 610 nm. Protein levels were calculated using standard bovine serum solution 200 mg in 100 ml of distilled water.

### Lipid peroxidation:

Lipid peroxidation was estimated by the method[33]. Liver homogenate mixed (1.0 ml) with 100 μl of 8.1% sodium dodecyl sulfate (SDS), and 600 μl of 20% acetic acid solution was kept for 2 min at room temperature, then 600 μl of 0.8% solution of TBA, was added, heated at 95° for 60 min in water bath and cooled with ice cold water at 4°. The mixtures of *n*-butanol, and pyridine (15:1, v/v) were added, shaken vigorously and centrifuged at 10.000 rpm for 5 min. The absorbance of the organic layer was measured at 532 nm. Lipid peroxidation was expressed as nmoles of MDA/mg of protein.

### Superoxide dismutase:

The SOD was estimated by the method[34,35], based on the reduction of Nitro blue tetrazolium (NBT) to water insoluble blue formazan. Liver homogenate (0.5 ml) was taken, and 1 ml of 50 mM sodium carbonate, 0.4 ml of 24 μm NBT, and 0.2 ml of 0.1 mM EDTA were added. The reaction was initiated by adding 0.4 ml of 1 mM hydroxylamine hydrochloride. Zero time absorbance was taken at 560 nm followed by 5 min at 25°. The control was simultaneously run without liver homogenate. Units of SOD activity were expressed as the amount of enzyme required to inhibit the reduction of NBT by 50%. The specific activity was expressed in terms of units/mg of protein.

### Catalase:

Catalase was estimated using a previously reported method[36]. The reaction mixture contained 1.0 ml of 0.01 M Phosphate buffer (pH 7.0), 0.1 ml of liver homogenate, and 0.4 ml of 2M H_2_O_2._ The reaction was stopped by the addition of 2.0 ml of dichromatic-acetic acid reagent. The control was carried out without addition of H_2_O_2,_ and then absorbance was read at 620 nm. CAT activity was expressed as μM of H_2_O_2_ consumed/min/mg protein.

### Statistical analysis

Statistical analysis was performed (GraphPadPrism 5 software, Inc) by using (Unpaired) student *t* test.

## RESULTS AND DISCUSSION

Liver homogenate of treated, and control rat was used for estimation of LPO, SOD, and CAT. The values observed were expressed as mean±SEM, using unpaired student *t* test as shown in Table 1. The blood serum of treated, and control rats was used to estimate the SGPT, SGOT, Alk Phos, cholesterol, and triglyceride as shown in Table 2. The values obtained clearly reveal that, LPO, SGPT, SGOT, Alk Phos, cholesterol and triglyceride were significantly (P<0.001) reversed by administration of *Calotropis gigantea* extracts in STZ-induced diabetic rats for 27 d. Consequently, the levels of SOD, and CAT were significantly (P<0.001) improved after administration of *Calotropis gigantea* extracts in STZ-induced diabetic rats.

**TABLE 1:** EFFECT OF *CALOTROPIS GIGANTEA* LEAF AND FLOWER EXTRACTS ON LPO, SOD, AND CAT LEVELS IN STZ-INDUCED DIABETIC RATS

Treatment	LPO MDA (nmol/mg protein)	SOD (Unit/mg protein)	Catalase (Unit/ mg protein)
Diabetic Control^a^	19.73±2.05***	9.81±0.24***	149.4±14.5***
Gliben^b^10 mg/kg	9.13±2.23**	14.52±0.70***	314.5±24.1***
Normal^c^	7.48±1.04	18.42±1.59	321.1±22.5
CLCH^d^ 10 mg/kg	11.49±0.84**	13.86±1.37*	294.5±42.8**
CLCH^d^ 20 mg/kg	13.12±0.88*	15.75±1.44**	288.9±34.5**
CLCH^d^ 50 mg/kg	11.99±0.80**	14.83±0.40***	239.5±0.2**
CFCH^e^ 10 mg/kg	12.20±1.18**	14.03±0.66***	228.6±19.5**
CFCH^e^ 20 mg/kg	12.17±1.09**	14.04±0.71***	218.2±22.4*
CFCH^e^ 50 mg/kg	12.96±1.29*	14.82±0.93***	219.0±18.6*

a Diabetic control: 5% Tween-80;

b glibenclamide;

c normal 5% Tween-80;

d *Calotropis gigantea* leaf chloroform;

e *Calotropis gigantea* flower chloroform extract. Values are expressed as mean±SEM, n=6 in each group

*** *P*<0.001, when (Unpaired t test) compared to diabetic control.

**TABLE 2:** EFFECT OF *CALOTROPIS GIGANTEA* LEAF AND FLOWER EXTRACTS ON SERUM BIOCHEMICAL PARAMETERS IN STZ-INDUCED DIABETIC RATS

Treatment	SGPT (U/dl)	SGOT (U/dl)	Alk Phos (U/dl)	Cholesterol (U/dl)	Triglycerides (U/dl)
Diabetic control^a^	256.80±21.29***	318.0±26.7***	238.5±9.0***	114.5±4.2**	168.0±9.4**
Gliben^b^ 10 mg/kg	68.57±6.52***	119.8±6.1***	186.9±10.8**	94.71±2.91**	122.2±9.2**
Normal^c^	60.99±2.32	64.6±4.67	127.4±2.2	84.36±3.86	115.1±6.8
CLCH^d^ 10 mg/kg	144.80±5.96***	197.6±5.6**	189.3±19.6*	97.75±5.04*	138.6±4.4*
CLCH^d^ 20 mg/kg	151.60±13.03**	167.4±7.4***	182.9±19.3*	97.92±2.75**	128.5±3.3**
CLCH^d^ 50 mg/kg	129.50±21.91**	172.7±14.6***	187.5±6.6**	96.66±3.22**	134.3±5.9*
CFCH^e^ 10 mg/kg	65.25±2.91***	158.6±2.8***	176.1±15.1**	95.23±3.94**	134.1±3.7**
CFCH^e^ 20 mg/kg	66.16±5.26***	150.2±8.5***	102.7±5.1***	88.71±4.34**	133.6±2.9**
CFCH^e^ 50 mg/kg	67.61±2.42***	165.5±3.8***	134.9±12.1***	92.50±3.74**	131.8±5.0**

a Diabetic control 5% Tween-80;

b glibenclamide;

c normal 5% Tween-80;

d *Caiotropis gigantea* leaf chloroform;

e *Caiotropis gigantea* flower chloroform extract. Values are expressed as mean±SEM, n=6 in each group

*** *P*<0.001, when (Unpaired t test) compared to diabetic control.

In order to establish a scientific basis for the utility of *Calotropis gigantea* in the treatment of diabetes, it was decided to evaluate free radical scavenging activity in STZ-induced diabetic rats. Earlier reports reveal that STZ-induced diabetic animals may exhibit most of the diabetic complication mediated through oxidative stress[37]. Glibenclamide is often used as an insulin stimulant in many studies, and also used as a standard antidiabetic drug in STZ-induced moderate diabetes to compare the antidiabetic properties of a variety of hypoglycemic compounds[37,38]. The findings of our study shows that protection provided by *Calotropis gigantea* extracts could maintain the levels of LPO, SOD, CAT, biomarker enzymes, cholesterol and triglycerides in STZ-induced diabetic rats.

Oxidative stress in diabetes is coupled to a decrease in the antioxidant status, which can increase the deleterious effects of free radicals[39]. The SOD and CAT are the two major scavenging enzymes that remove free radicals *in vivo*[40]. A decreased activity of these antioxidants can lead to an excess availability of superoxide anion (O_2_^˙־^) and hydrogen peroxide (H_2_O_2_), which in turn generate hydroxyl radicals (^·^OH), resulting in initiation and propagation of LPO. The SOD can catalyze dismutation of (O_2_^˙־^) into H_2_O_2_, which is then deactivated to H_2_O by catalase or SOD works in parallel with selenium-dependent glutathione peroxidase, which plays an important role in the reduction of H_2_O_2_ in the presence of reduced glutathione forming oxidized glutathione, and it protects cell protein and cell membranes against oxidative stress[40,41]. In our study, the SOD and CAT enzymes were significantly (P<0.001) decreased in STZ-induced diabetic control rats, may be due to inactivation caused by free radicals.

However, administration of *Calotropis gigantea* extracts on LPO levels were significantly reduced by both leaf and flower extract at oral dose (10 mg/kg). The flower extract oral dose (10 mg/kg) has restored SOD levels comparable with positive control, where as leaf extract has shown similar activity at oral dose (50 mg/kg). Where as, there is no significant change on CAT levels neither by leaf nor flower extract even at higher dose.

The SOD and CAT play a prominent role in scavenging free radical and restoring antioxidant activities in the tissue of diabetic animals[42–44]. The above observations may clearly suggest that increased levels of SOD and CAT of *Calotropis gigantea* extracts has free radical scavenging activity, which may exert a beneficial effect against pathological alterations caused by reactive oxygen species.

The elevation of biomarker enzymes, such as SGPT, SGOT and Alk Phos was observed in diabetic control rats indicating the hepatic damage[45]. The hepatic damage was restored and the transaminases were significantly reduced by flower *Calotropis gigantea* extracts, while leaf extract has less influence, subsequently Alk phos significantly reduced in flower extract at oral dose (20 and 50 mg/kg) than leaf extract at all doses as comparable to positive control. The diabetic complications such as increased gluconeogenesis and ketogenesis may be due to elevated transaminases activity[46]. Diabetic control rats show an important lipolytic activity, due to the insulinopenic state which contributes to maintain the abnormally elevated serum cholesterol and triglyceride levels in STZ-induced diabetic rats[47]. There is significant decrease in serum cholesterol and triglyceride levels in *Calotropis gigantea* extracts treated rats as evident by reports exhibiting a potent hypocholesterolemic effect. However, the possible underlying mechanism is not elucidated at this stage of the study. The previous studies have reported that administration of *Momordica charantia* lead to decrease in cholesterol levels probably by two mechanisms, one by decreasing absorption of cholesterol from intestine by binding with bile acids within intestine and increasing the extraction of faecales bile acids and the other by biosynthesis of cholesterol especially by decreasing the activity of 3-hydroxyl-3-methly-glutaryl coenzymes A reductase (HMG CoA reductase) an enzyme of cholesterol biosynthesis[47]. Same mechanism may be appropriate to explain the observed cholesterol and triglycerides lowering activity by *Calotropis gigantea* extracts.

The qualitative chemical test of the crude extract shows (Table 3) the presence of alkaloids, and flavanol glycoside supporting the earlier studies of *Calotropis gigantea* extracts[12,14,23,26]. Flavonoids are a group of naturally occurring compounds widely distributed as secondary metabolite in the plant kingdom[48]. One of the flavonoid glycosides prevent oxidant injury and cell death by several mechanism, such as, scavenging oxygen free radicals, protecting antioxidant enzymes, lipid peroxidation[11,48,49].

**TABLE 3:** PHYTOCHEMICAL ANALYSIS OF *CALOTROPIS GIGANTEA*

Chemical components	Leaf	Flowers
Alkaloids	+	+
Carbohydrate	−	−
Phytosterols	−	−
Glycosides	+	+
Flavonoids	+	+
Tannins	+	+
Proteine and amino acids	−	−
Saponins	−	−
Vitamine C	−	−

In the present study, it is concluded that *Calotropis gigantea* extract has free radical scavenging activity and improved antioxidant effect. The precise mechanism(s) and site(s) of action as well as constituents of *Calotropis gigantea* will be further determined including their toxicological effects.

## References

[1] Halliwell B, Gutteridge JM (1989). Free radicals in biology and medicine.

[2] Halliwell B, Gutteridge JM (1990). Role of free radicals and catalytic metal ions in human disease. Met Enzymol.

[3] Halliwell B, Gutteridge JM (1994). Lipid peroxidation, oxygen radicals, cell damage and antioxidant therapy. Lancet.

[4] Simmons KJ (1984). Defense against free radicals has therapeutic implications. J Am Med Assn.

[5] Wohaieb SA, Godin DV (1987). Alterations in free radical tissue defense mechanism in STZ-induced diabetes in rat, effects of insulin treatment. Diabetes.

[6] McLennan S, Heffermen V, Wright S (1991). Change in hepatic glutathione metabolism in diabetes. Diabetes.

[7] Tarique A, Sharma M, Pillai KK, Haque SE, Alam MM, Zaman MS (2007). Protective effect of bezfibrate on streptozotocin-induced oxidative stress and toxicity in rats. Toxicol.

[8] Mitra A, Chakraborty S, Auddy B, Tripathi P, Sen S, Saha AV (2002). Evaluation of chemical constituents and free-radical scavenging activity of Swarnabhasma (gold ash) an ayurvedic drug. J Ethnopharmacol.

[9] Srivastava Y, Bhat HV, Verma Y, Venkaidh K (1993). Antidiabetic and adaptogenic properties of *Momordica charantia* extract: An experimental and clinical evaluation. Phytother Res.

[10] Rao Rao BK, Rao CH (2001). Hypoglycemic and antihyperglycemic activity of *Alternifolium* (Wt) Walp. seed extracts in normal and diabetic rats. Phytomedicine.

[11] Roy S, Sehgal R, Pahdy BM, Kumar VL (2005). Antioxidant and protective effect of *Calotropis procera* against alloxan-induced diabetics in rats. J Ethnopharmacol.

[12] Rathod N, Raghuveer I, Chitme HR, Chandra R (2009). Prevention of high-fructose diet induced insulin resistance by *Nyctanthes arbortristis* and *Calotropis gigantea* in rats. Phcog Mag.

[13] Lodhi G, Singh HK, Pant K, Hussain Z (2009). Hepatoprotective effect of *Calotropis gigantea* extract agaist carbon tetrachloride induced liver injury in rats. Acta Pharma.

[14] Chitme HR, Chandra R, Kaushik S (2004). Studies on anti-diarrhoeal activity of *Calotropis gigantea* R. Br. in experimental animals. J Pharm Pharm Sci.

[15] Chitme HR, Chandra R, Kaushik S (2005a). Evaluation of antipyretic activity of *Calotropis gigantea* (Asclepiadaceae) in experimental animals. Phytother Res.

[16] Chitme HR, Chandra R, Kaushik S (2005b). Evaluation of analgesic activities of *Calotropis gigantea* extract *in vivo*. Asia Pac J Pharmacol.

[17] Adak M, Gupta JK (2006). Evaluation of anti-inflammatory activity of *Calotropis gigantea* (AKANDA) in various biological systems. Nepal Med Coll J.

[18] Argal A, Pathak AK (2006). CNS activity of *Calotropis gigantea* roots. Ethnopharmacogical communication. J Ethnopharmacol.

[19] Deshmukh PT, Fernandes J, Atul A, Toppo E (2009). Wound healing activity of *Calotropis gigantea* root bark in rats: Ethnopharmacogical communication. J Ethnopharmacol.

[20] Srivastava SR, Keshri G, Bhargavana B, Singh C, Singh MM (2007). Original research article: Pregnancy interceptive activity of the roots of *Calotropis gigantea* Linn. in rats. Contraception.

[21] Pathak AK, Argal A (2007). Analgesic activity of *Calotropis gigantea* flower. Fitoterapia.

[22] Rajesh R, Raghavendra Gowda CD, Nataraju A, Dhananjaya BL, Kemparaju K (2005). Procoagulant activity of *Calotropis gigantea* latex associated with fibrin(ogen)olytic activity. Toxicon.

[23] Thakur S, Das P, Itoh T, Kazunori I, Taro M (1984). Latex extractable of *Calotropis gigantea*. Phytochemistry.

[24] Thitima L, Somyot S (2006). 19-Nor-and 18,20-Epoxy-cardenolides from the leaves of *Calotropis gigantea*. J Nat Prod.

[25] Rowshanul HM, Farjana N, Matiar R, Ekramul HM, Rezaul KM (2007). Isolation of stigmasterol and ß-sitosterol from methnolic extract of root bark of *Calotropis gigantea*. Pak J Bio Sci.

[26] Sen S, Niranjan PS, Mahato SB (1992). Flavonol glycosides from *Calotropis gigantea*. Phytochemistry.

[27] Sengupta A, Bhattacharya D, Pal G, Sinha NK (1984). Comparative studies calotropin DI and DII from the latex of *Calotropis gigantea*. Arch Biochem Biophys.

[28] Heinemann U, Pal GP, Hilgenfeld R, Saenger W (1982). Crystal and molecular structure of the sulfhydryl protease calotropin DI at 3.2 A° resolution. J Mol Biol.

[29] Babu V, Ganadevi T, Subramoniam A (2003). Antidiabetic activity of ethanol extract of *Cassia Klenii* leaf in streptozotocin-induced diabetic rats and isolation of an active fraction and toxicity evaluation of the extract. Indian J Pharmacol.

[30] Rathod N, Raghuveer I, Chitme HR, Chandra R (2008). Antidiabetic activity of *Nyctanthes arbortristis* Phcog Mag.

[31] Purohit A, Sharma A (2006). Blood glucose lowering potential of *Bougainvillea spectabilis Willd*. leaf extract in streptozotocin-induced type-1 diabetics albino rats. Indian Drugs.

[32] Lowry OH, Rosebrough NJ, Farr AL, Randall RJ (1951). Protein measurement with the Folin phenol reagent. Biol Chem.

[33] Ohkawa H, Ohishi N, Yagi K (1979). Assay for lipid peroxides in animal tissues by thiobarbituric acids reaction. Anal Biochem.

[34] Beauchamp C, Fridovich I (1971). Superoxide dismutase: Improved assays and an assay applicable to acrylamide gels. Anal Biochem.

[35] Chidambara Murthy KN, Jayaprakasha GK, Singh RP (2002). Studies on antioxidant activity of Pomegranate (*Punica granatum*) peels extract using *in vivo* models. J Agri Food Chem.

[36] Sinha A (1972). Calorimetric assay of catalase. Anal Biochem.

[37] Sathishsekar D, Subramanian S (2005). Antioxidant properties of *Momordica charantia* (bitter gourd) seeds on streptozotocin-induced diabetic rats. Asia Pac J Clin Nutri.

[38] Venkatesh S, Reddy GD, Reddy YS, Satayavathy D, Reddy BM (2004). Effect of *Helicteres isora* root on glucose tolerance in glucose induced hyperglycemic rats. Fitoterapia.

[39] Picton SF, Flatt PR (2001). Differential acute and long term actions of succinic acid monomethyl ester exposure on insulin secreting BRAIN-BN 11 cells. Int J Exp Diabetes Res.

[40] Jin L, Xue HY, Jin LJ, Li SY, Xu YP (2008). Antioxidant and pancreas-protective effect of aucubin on rats with streptozotocin-induced diabetes. Eur J Pharmacol.

[41] Aebi H (1984). Catalase *in vitro*. Met Enzymol.

[42] Tuzun S, Girgin FK, Sozmen EY (1999). Antioxidant status in experimental type 2 diabetes mellitus: Effect of glibenclamide and glipizide on various rat tissue. Exp Toxicol Pathol.

[43] Elmali E, Altan N, Bukan N (2004). Effect of the sulphonylurea glibenclamide on liver and kidney antioxidant enzymes in streptozotocin-induced diabetic rats. Drugs RD.

[44] Rahimi R, Nikfar S, Larijan B, Abdollahi M (2005). A review on the role of antioxidant in the management of diabetes and its complications. Biomed Pharmacother.

[45] Selvan VT, Manikandan L, Senthil Kumar GP, Suresh R, Kakoti BB, Gomathi P (2008). Antidiabetic and antioxidant effect of methanol extract of *Artanema sesamoides* in streptatozocin-induced diabetic rats. Int J Appl Res Nat Prod.

[46] Ghosh S, Suryawansi SA (2001). Effect of *Vinca rosea* extracts in treatment of alloxan diabetes in male albino rats. Indian J Exp Biol.

[47] Lemhadri A, Hajji L, Michel JB, Eddouks M (2006). Cholesterol and triglyceride lowering activity of caraway fruits in normal and streptozotocin diabetic rats. J Ethnopharmacol.

[48] Omer C, Mehmet K, Ahmet K, Sukru O (2005). Quercetin, a flavonoid antioxidant, prevents and protects streptozotocin-induced oxidative stress and β-cell damage in rats pancreas. Pharmacol Res.

[49] Abraham LCN, Masakuni T, Isao H, Hajime T (2008). Antioxidant flavonoid glycosides from the leaves of *Ficus pumila* L. Food Chem.

